# Competency of groundwater recharge of irrigated cotton field subjacent to sowing methods, plastic mulch, water productivity, and yield under climate change

**DOI:** 10.1007/s11356-021-17017-0

**Published:** 2021-10-21

**Authors:** Muhammad Saeed, Ahsan Maqbool, Muhammad Adnan Ashraf, Muhammad Arshad, Kashif Mehmood, Muhammad Usman, Muhammad Arslan Farid

**Affiliations:** 1 Department of Irrigation and Drainage, University of Agriculture Faisalabad, Faisalabad, 38000 Pakistan; 2 Institute for Sustainable Agriculture, Spanish National Research Council, 14001 Cordoba, Spain; 3 Center for Development Research (ZEF), University of Bonn, 53113 Bonn, Germany; 4 Department of Geoecology, Institute of Geosciences and Geography, University of Halle-Wittenberg, 06120 Halle (Saale), Germany

**Keywords:** Groundwater, Recharge flux, Climate change, HYDRUS-1D, Cotton

## Abstract

Irrigated agriculture is a foremost consumer of water resources to fulfill the demand for food and fiber with an increasing population under climate changes; cotton is no exception. Depleting groundwater recharge and water productivity is critical for the sustainable cotton crop yield peculiarly in the semiarid region. This study investigated the water productivity and cotton yield under six different treatments: three sowing methods, i.e., flat, ridge, and bed planting with and without plastic mulch. Cotton bed planting without mulch showed maximum water productivity (0.24 kg.m^−3^) and the highest cotton yield (1946 kg.ha^−1^). Plastic mulching may reduce water productivity and cotton yield. HYDRUS-1D unsaturated flow model was used to access the groundwater recharge for 150 days under six treatments after model performance evaluation. Maximum cumulative recharge was observed 71 cm for the flat sowing method without plastic mulch. CanESM2 was used to predict climate scenarios for RCP 2.6, 4.5, and 8.5 for the 2050s and 2080s by statistical downscale modeling (SDSM) using historical data from 1975 to 2005 to access future groundwater recharge flux. Average cumulative recharge flux declined 36.53% in 2050 and 22.91% in 2080 compared to 2017 without plastic mulch. Multivariate regression analysis revealed that a maximum 23.78% reduction in groundwater recharge could influence future climate change. Further study may require to understand the remaining influencing factor of depleting groundwater recharge. Findings highlight the significance of climate change and the cotton sowing method while accessing future groundwater resources in irrigated agriculture.

## Introduction

Irrigated agriculture is a foremost consumer of water resources to fulfill the demand for food and fiber with an increasing population under climate changes. Insufficient water to keep plant cells hydrated substantially limits all crop species’ productivity. Cotton production is certainly no exception; globally, 250 billion tons of water annually are required. The water footprint from cotton consumption of developed countries is cross-border, highest impact in Pakistan, India, China, and Uzbekistan (Chapagain et al. 2006). Cotton crop provides fiber to the textile industry, fulfills nutrition demands in edible canola oil, and is used as herbal medicine in ancient to modern science (Shahrajabian et al. 2020). Improving water productivity can enhance cotton yield in arid to semiarid regions sensitive to climate risk (Chen et al. 2020; Abbas 2020). Rising temperature decreases cotton yield while also exerting additional stress on water demands for the cotton that lead to a risk of socio-economic development in the agro-ecological zone (Naheed and Rasul 2005; Abbas 2020; Saleem et al. 2021). Sacred surface water has posed significant challenges for sustainable development to the agricultural sector at a local level (Mikosch et al. 2020). In the semiarid region of Pakistan, groundwater plugs 56% of crop water requirements (Maqbool et al. 2021). Groundwater levels are declining up to 1.3 m.year^−1^ due to pumping (Khaliq et al. 2021) and have dropped approximately 25 m (Khan et al. 2017). A substantial depletion in groundwater level is expected for 2036–2045 due to increased crop water consumption under changing climate (Usman et al. 2020). Socio-economic and environmental sustainability are narrowly interweaved. These are necessary gears for sustainable agriculture (Brodt et al. 2011). l. 2011). Moreover, achieving food security is only possible through better understanding the complexity of the agricultural system and re-design practices. In response to climate change, it is essential to assess groundwater recharge in an irrigated cotton field to adopt a mitigation strategy that improves water productivity and cotton yield for rural socio-economic development.

Groundwater recharge is a bouncy source of freshwater that crosses the aquifer from surface to subsurface water ponding via terrestrial infiltration (Anderson et al. 2015). Estimating groundwater recharge is one of the provoking duties due to hydrologic and environmental applications; vulnerability is attributed to climate change (Şen 2015; Usman et al. 2020), becoming more sensitive to irrigated crops (Ficklin et al. 2010; Hama et al. 2020). Increasing temperature (5.7 °C annually by the late twenty-first century) is declining rainfall (11.9% in the mid-twenty-first century) (Dahri et al. 2021) that is strongly related to groundwater recharge (Pool et al. 2021a). Irrigated (paddy) field has a high potential for percolation of water over 30 mm.day^−1^ that could be increased by efficient irrigation and crop management practices (Hama et al. 2020). On the contrary, irrigation-related recharge could be problematic where excessive recharge or insufficient groundwater abstraction may raise the water table, creating waterlogged and salinity land (Owen 2021; Maqbool et al. 2021). In addition, the input of irrigation and fertilizer may alter groundwater recharge fluxes and mobility of salts/pollutants from unsaturated zone to groundwater (Turkeltaub et al. 2014). It stimulates concrete measures required to retain aquifer sustainability in irrigated Indus Basin region (Cheema et al. 2014). Nonetheless, evaluating groundwater recharge is crucially essential to the sustainability of groundwater supplies. That is exceptionally susceptible to human influences due to pervasive land uses.

Crop and hydrologic models can access water variability in agro-hydrologic development themes for groundwater recharge (Siad et al. 2019). Hydrological models are emphasized more due to mechanistic processes of soil water movement (Tenreiro et al. 2020). HYDRUS-1D can simulate the solute movement in the vadose zone and recharge flux below the root zone (Šimůnek et al. 2013). It can develop a vertical unsaturated flow model and reported a 44% drop in groundwater recharge by agricultural (orchard and crop) fields under changing climate (Turkeltaub et al. 2014). Water use efficiency could reduce groundwater recharge as increased daily temperatures from 1.1 to 6.4 °C (Ficklin et al. 2010). Recharge flux would decrease by 0.0.090 to 0.0.210 m using HYDRUS-1D for the semiarid region (Kambale 2017). Nevertheless, the HYDRUS-1D model could provide better insight into groundwater recharge simulation, including capillary rise, hysteresis, preferential flow, subsurface lateral flow, and surface outflow, for spatial water processes (Tenreiro et al. 2020). Furthermore, statistical downscale modeling (SDMS) is a reliable decision support tool to generate future climatic parameters based on historical trends in general circulation models (GCMs) (Usman et al. 2020; Saleem et al. 2021) for a potential climate-driven impact assessment on groundwater recharge (Azam et al. 2020; Wu et al. 2020). Since 60% of irrigation water is lost due to poor conveyance infrastructure and inadequate application in the field (Imran 2019). This eventually leads to reduction in crop performance. The sowing method could be an appropriate option to access groundwater recharge in addition to water productivity and yield (Ficklin et al. 2010; Shah et al. 2016).

It is essential to look at an irrigated cotton field because climate change is not homogenous across the Indus Basin region. Different strategies need to adopt to increase cotton yield and water use efficiency to mitigate their impact. Therefore, this study was designed to investigate the following objective: (1) evaluate the water productivity and cotton yield under six different treatments (three sowing methods, i.e., flat, ridge, and bed planting with and without plastic mulch); (2) develop, calibrate, and validate the unsaturated flow model HYDRUS-1D to simulate groundwater recharge from irrigated cotton fields under six different treatments; and (3) access the impact of future climate change on groundwater recharge under six different treatments to do this historical data from 1975 to 2005 of climatic parameters, i.e., minimum and maximum temperature, rainfall, relative humidity, and wind speed, used in SDMS to predict their variability for future 2021–2050 and 2051–2080 under representative concentration pathway (RCP) 2.6, RCP 4.5, and RCP 8.5, respectively. Meantime, a relationship of groundwater recharge and climatic parameters was also quantified by performing regression and correlation analysis.

## Materials and methods

### Study area

**Fig. 1:**
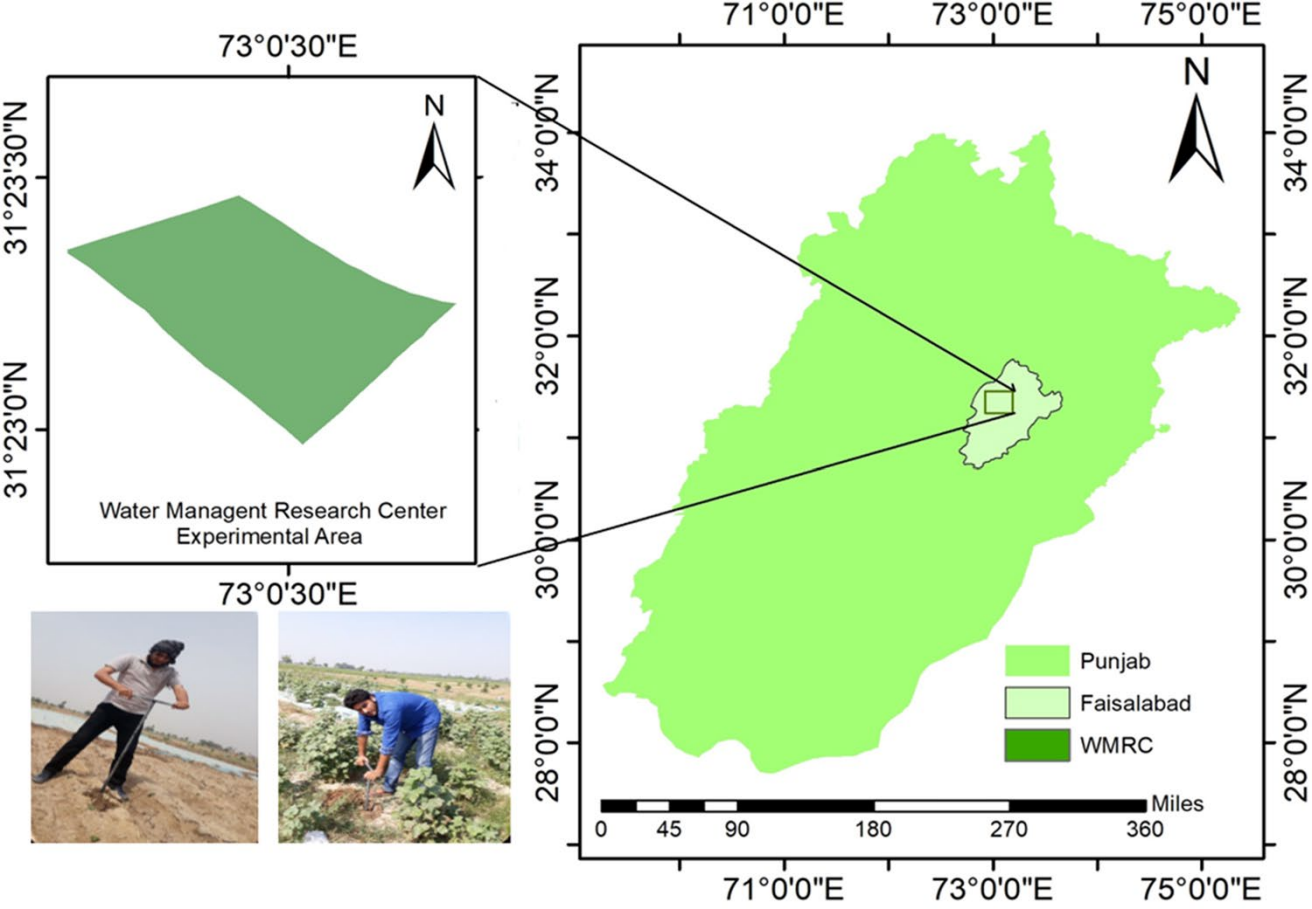
Cotton irrigated field sampling sites at Water Management Research Center (WMRC), Faisalabad

The study was conducted under a cotton (*Gossypium*) field, located in Rechna Doab upland of Punjab at Water Management Research Center (WMRC), Faisalabad (31.3872° N latitude and 73.0121° E longitude, Fig. 1). This area has a semiarid climate in Köppen-Geiger classification, with sweltering and humid summers (April–October) and dry, cool winters (November–March). The maximum temperature in the hottest month is 50℃ (June). The minimum temperature in the coldest month is 8℃ (December), an altitude of 184 m above mean sea level based on the nearest (under 2 km of research site) station’s data provided by that National Institute for Agriculture and Biology (NIAB), Faisalabad. The average rainfall in the area is 283 mm, and the maximum rainfall event occurred during July. The study area falls in a mixed cropping zone of the Punjab agro-ecological zone, including wheat, rice, cotton, sugarcane, and maize that are to be grown (AMIS 2016; Saleem et al. 2021). The cropping intensity of the Faisalabad district now reaches ~ 150% due to the high demand for food (edible) crops. The study area part of the Indus plain that comes under the area has fertile land due to the alluvial soil genesis of Chenab Delta. The soil type is medium texture and homogenous structure up to 4 m depth. This plain is underlain by thick sandy loam to loamy sand with high hydraulic conductivity (Kelleners et al. 1999). According to the soil survey, the site’s soil has low organic matter and a slightly base pH (7.0–7.9), which is feasible for various crops.

### Experimental setup and data collection

Randomized complete block design (RCBD), the sowing method, and land cover experimented with three sowing methods were studied, i.e., flat sowing, ridge sowing, and bed sowing with mulch and non-mulch land cover. There were six treatments, i.e., T_1_ (flat sowing with mulch), T_2_ (flat sowing without mulch), T_3_ (ridge sowing with mulch), T_4_ (ridge sowing without mulch), T_5_ (bed planting with mulch), and T_6_ (bed planting without mulch), respectively, with three repetitions. Plastic film with low-density polyethylene (LDPE) is used as mulching. The plot size was 6 m × 4.5 m, and the total experimental area was about 0.141 ha. The cotton crop was sown on June 13, 2017, and harvested on November 20, 2017, with three replications of each treatment. Cottonseeds were planted at 25 kg.ha^−1^ on edges by keeping up 75 cm row to row. Two seeds for every slope were dibbled to a profundity of 3 to 4 cm by separating 30 cm from plant to plant in a row to achieve the required plant population. Weeds management strategy was adopted from early season to harvesting.

Moisture content data was collected from the fields at 7-day intervals. The soil sampling was done at 0–30 cm, 30–60 cm, and 60–90 cm depth from each treatment plot. A fixed interval soil sample was collected using a vertical borehole (auger-hole) drilling method (Fig. 1). The moisture content of the soil sample was measured using the oven-dry method for 24 h at 105℃. Gravimetric moisture content ${(\uptheta }_{\mathrm{g}})$ was obtained, and volumetric moisture content ${(\uptheta }_{\mathrm{v}})$ was calculated by ${\uptheta }_{\mathrm{v}}=$ ${\uptheta }_{\mathrm{g}} \times \text{apparent specific gravity}.$ Meanwhile, apparent specific gravity is the ratio of ${\rho }_{s}$= bulk density of soil (g/cm^3^) and ${\rho }_{w}$= bulk density of water (g/cm^3^). In addition, climatic data of the Faisalabad station was acquired from Pakistan Metrological Department (PMD).

**Fig. 2:**
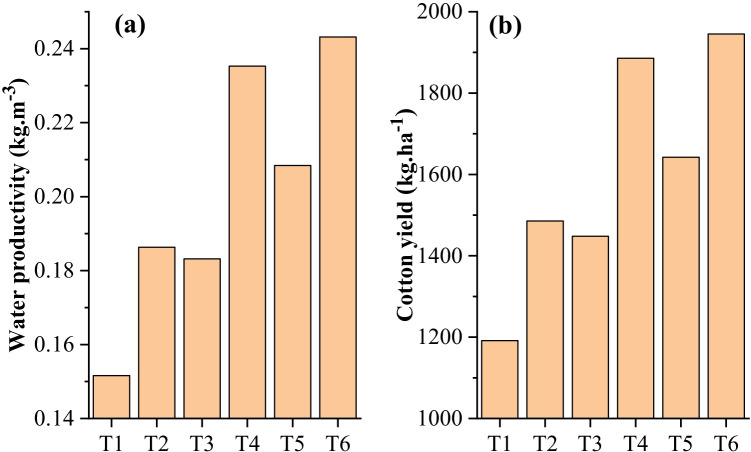
Experimentally measured **a** water productivity and **b** cotton yield in six different treatments

### Fertilization, water productivity, and cotton yield

The recommended dosage of phosphorus (100 kg.acre^−1^) in the form of DAP (di-ammonium phosphate), potash (75 kg.acre^−1^) in the form of sulfate of potash, and nitrogen (140 kg.acre^−1^) in the form of urea was used at the time of seeding as per standard guidelines of the Extension and Adaptive Research Wing (EARW), Punjab Agriculture Department. Nitrogen (35 kg.acre^−1^) in urea was applied after 30 days of sowing, during boll formation initiation, and boll formation completion. Irrigation water was applied as a rotational program followed by EARW, with fixed irrigation interval based on the sowing method. The irrigation interval is 7 days for bed and ridge sowing and 10 days for flat sowing cotton. Irrigation depth was calculated by Eq. (1):

1 $$QT=AD$$

where *Q* is discharge of the outlet/tube well (cusec), *T* is time of irrigation (hour), *A* is area to be irrigated (acre), and *D* is depth of applied irrigation (inch).^1^

Water productivity (WP, kg.m^−3^) was measured for each treatment using Eq. (2):

2 $$WP=\frac{cotton yeild }{total water applied}$$

Cotton yield per hectare is calculated by picking cottonseed from each plant in all six plots and weighted.

### HYDRUS-1D: estimation of recharge flux

Unsaturated flow models were developed for each treatment, i.e., T_1_, T_2_, T_3_, T_4_, T_5_, and T_6_, respectively, to simulate groundwater recharge flux. Richard’s convection–dispersion equations were espoused to account for the water flow under the cotton field. HYDRUS-1D code was run to simulate the one-dimensional flow based on Eq. (3) (Šimůnek et al. 2013):

3 $$\frac{\partial \theta }{\partial t} =\frac{\partial }{\partial z}\left[K\left(h\right)\frac{\partial h}{\partial z}+K(h)\right]-S(h)$$

where θ is the volumetric soil water content (cm^3^.cm^−3^), *t* is time (day), *h* is the soil water pressure head (cm), *z* is the gravitational head as well as the vertical coordinate (cm, upward is positive), *K*(*h*) is the unsaturated hydraulic conductivity (cm.day^−1^), and *S* is the soil water extraction rate by plant roots (cm^3^.cm^−3^.day^−1^).

The soil hydraulic properties of soil were elucidated by the Mualem-van Genuchten formulation (Mualem 1976; van Genuchten 1980), based on the pore-size distribution in the model by Eqs. (4) and (5), respectively:

4 $${S}_{e}= \frac{\theta -{\theta }_{r}}{{\theta }_{s}-{\theta }_{r}}= {\left(1+ {\alpha }_{r} {\left|h\right|}^{n}\right)}^{-m}$$

5 $$K\left(h\right)={K}_{s}{{S}_{e}}^{l}{\left[1-{\left(1-{{S}_{e}}^{1/m}\right)}^{m}\right]}^{2}$$

where *S*_*e*_ is effective saturation [-]; θ_r_ and θ_s_ are residual and saturated water contents [cm^3^.cm^−3^], respectively; and *α*_r_ (cm^−1^), m [-], and n [-] are shape parameters. *K*_*s*_ is the saturated hydraulic conductivity (cm.day^−1^); l [-] is a pore connectivity parameter; and m = 1–1/n, when *n* > 1. The percentage of sand, silt, and clay and bulk density and van Genuchten parameters are given in Table 1.

**Table 1:** Soil properties of experimental site including particle-size distribution at different depths by weight percentage of particle-diameter intervals (sand, silt, and clay) and van Genuchten parameters

Depth (cm)	Sand (%)	Silt (%)	Clay (%)	Bulk density (g cm^−3^)	*K*_*s*_ (cm.day^−1^)	θ_s_ (cm^3^.cm^−3^)	θ_r_ (cm^3^.cm^−3^)	*α* (cm^−1^)	*n*
0–30	74.25	12.35	13.40	1.660	106.1	0.0809	0.04	0.075	1.89
30–60	73.33	12.62	14.05	1.650	106.1	0.1401	0.05	0.075	1.89
60–90	73.5	12.80	13.75	1.642	106.1	0.1408	0.05	0.075	1.89

At first, the model’s domain and soil parameters were conventional taken into account by the measured particle-size distributions (Table 1) and monitored moisture contents at different depths (0–30, 30–60, and 60–90 cm), respectively. Subsequently, undergoing model calibration, soil parameters were slightly modified to best fit corresponding observations and model predictions.

### Flow model: boundary and initial conditions

The upper boundary condition was selected as atmospheric surface runoff that depended on evapotranspiration, irrigation, and rainfall. For this study, reference evapotranspiration (ET_0_) was estimated using the NIAB meteorological station’s recorded data by the Penman–Monteith equation. Meanwhile, the lower boundary condition was free drainage to count the root zone’s recharge flux. Water content was set as initial conditions to calculate the root water uptake (transpiration). The proposed method to include osmotic stress was applied as represented in Eq. 6 (van Genuchten 1987). In the current study, the multiplicative threshold model was used:

6 $$\alpha \left(h,{h}_{\phi }\right)=\frac{1}{1+{\left({~}^{h}\!\!\left/\!\!{~}_{{h}_{50}}\right.\right)}^{{P}_{1}}} \frac{1}{1+{\left({~}^{{h}_{\phi }}\!\!\left/\!\!{~}_{{h}_{\phi 50}}\right.\right)}^{{P}_{2}}}$$

where *p*_1_ and *p*_2_ are experimental constants, *h*_50_ is the pressure head at which the water extraction rate is reduced by 5% during conditions of negligible osmotic stress, and *h*φ_50_ is the osmotic head at which the water extraction rate is reduced by 50% during negligible water stress. The Feddes parameter values for root water uptake for the cotton crop were estimated given in Table 2.

**Table 2:** Root water uptake parameters for cotton crop

P.O. (cm): value of the pressure head below which roots start to extract water from the soil	− 10
POpt (cm): value of the pressure head below which roots extract water at the maximum possible rate	− 25
P2H (cm): value of the limiting pressure head below which roots can no longer extract water at the maximum rate (assuming a potential transpiration rate of *r2H*)	− 200
P2L (cm): as above, but for a potential transpiration rate of *r2L*	− 600
P3 (cm): value of the pressure head below which root water uptake ceases (usually taken at the wilting point)	− 14,000
r2H (cm/days): potential transpiration rate [LT^−1^] (currently set at 0.5 cm/day)	0.5
r2L (cm/days): potential transpiration rate [LT^−1^] (currently set at 0.1 cm/day)	0.1

The governing equation is the water balance of the vertical soil domain covered by vegetation for a given period. There was no runoff during irrigation. The cotton plants’ interception of rainwater was neglected due to low rainfall amounts during the vegetation period. FAO-56 standards calculated potential evapotranspiration (ETo) by the Penman–Monteith method. Metrological parameters that include solar radiation, relative humidity, and leaf area index are used for the growth period. Meanwhile, crop evapotranspiration (ET_c_) was computed by a regional crop coefficient (K_c_) based on the initial season, mid-season, late season, and evaporation soil. Evaporation from the soil is predicted by the estimation of energy available at the soil surface.

### Model calibration and validation

Model calibration is typically defined as turning a model by interpreting the input parameters, i.e., soil hydraulic parameters and boundary and initial conditions. The van Genuchten hydraulic parameters, i.e., *θ*_*r*_, *θ*_*s*_,$\alpha ,$ and *n*, were calibrated using the soil moisture content at three different depths in the root zone of an irrigated cotton field. Inverse estimation methods were used to determine unsaturated soils’ hydraulic properties, i.e., θ_(h)_ and K_(θ)_(Bitterlich et al. 2004). Meanwhile, pedotransfer functions were applied to predict water retention parameters and saturated hydraulic conductivity from soil texture and bulk density. These estimations were refined by induction of input data of water retention points (one or two). The optimal parameters were determined using a repetitive model operation approach attributed to the slightest difference between observed and predicted soil water content to site-specific conditions. For this purpose, soil moisture content, tension, and salinity data were measured every fifth day before and after irrigation. Then the model was validated using the calibrated model parameters for soil moisture content for different agronomical practices of irrigated cotton grown during replicate.

### Model performance criteria

The model performance was evaluated by the root mean square error (*RMSE*), coefficients of correlation (*R*^2^), and Nash–Sutcliffe efficiency (*NSE*) and calculated by Eqs. (7), (8), and (9) as follows:

7 $$RMSE=\sqrt{\frac{1}{2}\sum_{k=1}^{n}{({X}_{k}-{Y}_{k})}^{2}}$$

8 $${R}^{2}=\frac{\sum_{k=1}^{n}{({X}_{k}-\overline{X })}^{2}\bullet {({Y}_{k}-\overline{Y })}^{2}}{\sqrt{\sum_{k=1}^{n}{(X-\overline{X })}^{2}\bullet \sum_{i=1}^{n}{({Y}_{k}-\overline{Y })}^{2}}}$$

9 $$NSE=1-\frac{\sum_{k=1}^{n}{({Y}_{k}-{X}_{k})}^{2}}{\sum_{k=1}^{n}{({X}_{k}-\overline{X })}^{2}}$$

where *X* is the observed value, *Y* is simulated value removal, the *bar (-)* represents the mean value, and *n* is the number of samples.

### Climate change scenarios for HYDRUS-1D simulation

Climate change scenarios are considered for the estimation of recharge flux at a regional level. Downscaled by the statistical downscaling modeling (SDSM) approach, it is a hybrid tool that supports examining the impact of climate change. Here, CanESM2 climatic variables are used and acquired from https://climate-scenarios.canada.ca/, which has historical data of 26 climatic parameters from 1976 to 2005, respectively. Grid data 27 X and 44 Y of CanESM2 were downloaded from the above link. The data is accessible at a grid resolution of 2.8125° latitude × 2.8125° longitudes, respectively. Moreover, CanESM2 gives future climatic data under different representative concentration pathways (RCP) for three scenarios RCP 2.6, 4.5, and 8.5 from 2006 to 2100.

SDMS can be done in three steps. The first is predictor’s screening: the most relevant atmospheric parameters were chosen based on *p*-value, histograms, scatterplots, correlation matrix, and partial correlation with the assistance of the MLR model. A correlation matrix was preferred between predictands and CanESM2 predictors (Kobuliev et al. 2021). A predictor parameter was positively correlated and has a significant representation in the scatterplot and minimum *p*-value. NCEP (National Centers for Environmental Prediction) predictands were different for each parameter and selected based on *p*-value and partial *r*-value, respectively. Therefore, it was cautiously chosen for maximum (minimum) temperature, rainfall, relative humidity, and wind speed.

The second is calibration and validation: data for the calibration period from 1976 to 1995 is used as a base and simulates the daily maximum (minimum) temperature, rainfall, relative humidity, and wind speed for the validation period from 1996 to 2005 of NCEP and CanESM2 predictors (95% confidence level). For better agreement with the results, 100 ensembles were used to obtain the average. Evaluation results (Table 3) show that accuracy downscaling in the reproduction of climatic parameters is based on high *R*^2^ (> 0.96) and low *RMSE* (< 0.4) for both calibration and validation period, consequently. In addition, *NSE* (> 0.95 ~ 1.0) is elucidated the good efficiency or better fitting of data. Evaluation performance has shown better agreement than the previous study (Bessah et al. 2021).

**Table 3:** Evaluation of climatic parameters for calibration (1975–1995) and validation (1996–2005) phase

Climatic parameters	Calibration			Validation		
	*R*^2^	*RMSE*	*NSE*	*R*^2^	*RMSE*	*NSE*
Max. temperature	0.965	0.348	0.973	0.975	0.221	0.981
Min. temperature	0.997	0.033	0.996	0.961	0.365	0.979
Rainfall	0.976	0.343	0.884	0.959	0.376	0.879
Relative humidity	0.980	0.113	0.959	0.986	0.110	0.968
Wind speed	0.988	0.077	0.986	0.973	0.367	0.969

The third is scenario generator: it is used to simulate the results of future climate scenarios, i.e., RCP 2.6, RCP 4.5, and RCP 8.5, respectively. The future climate data of maximum (minimum) temperature, rainfall, relative humidity, and wind speed were predicted for 2006 to 2100 on a daily basis and studied for two periods, i.e., 2021 to 2050 and 2051 to 2080. The increasing trend of maximum (minimum) temperature (Table 4) is similar to previous reports lately (Arshad et al. 2019; Saddique et al. 2020). Moreover, rainfall, relative humidity, and wind speed will decline significantly, as their magnitude is presented in Table 4. These climate variables are influential in evaluating the blue water footprint under cotton fields (Huang et al. 2019), causing a diminution of groundwater recharge. Then, data was used to estimate the groundwater recharge flux under different sowing conditions for the irrigated cotton field.

**Table 4:** Predicted relative changes in average values for future climatic parameters under RCP 2.6, RCP 4.5, and RCP 8.5

Parameters	2021–2050			2051–2080		
	RCP 2.6	RCP 4.5	RCP 8.5	RCP 2.6	RCP 4.5	RCP 8.5
Max. temperature (℃)	2.03	2.11	2.18	3.40	3.64	4.04
Min. temperature (℃)	0.80	0.91	1.07	1.06	1.51	2.18
Rainfall (mm)	48.22	47.92	47.75	41.68	41.41	40.18
Relative humidity (%)	− 4.52	− 4.42	− 4.37	− 10.20	− 10.33	− 10.35
Wind speed (km/hr)	− 0.40	− 0.37	− 0.35	− 0.95	− 0.85	− 0.69

### Statistical analysis

Microsoft Excel® 2019 add-in data analysis tool was used to develop a correlation and multivariate regression model. The linear relationship between tended groundwater recharge and climate variables was established to determine the groundwater recharge flux.

## Results and discussions

### Water productivity and cotton yield

Water productivity plays a crucial role in sustainable agriculture, increasing yield production per unit of water used. Apparently (Fig. 2), the cotton sowing method without mulching (T_2_, T_4_, and T_6_) practice has proven to be more water-efficient than mulching (T_1_, T_3_, and T_5_). Cotton bed planting without mulch showed maximum water productivity (0.24 kg.m^−3^). Similarly, the cotton sowing method without mulching (T_2_, T_4_, and T_6_) has demonstrated high cotton yield than mulching (T_1_, T_3_, and T_5_). Meantime, cotton bed planting showed the highest cotton yield (1946 kg.ha^−1^), followed by ridge sowing without mulch (1886 kg.ha^−1^) and bed planting with mulch (1643 kg.ha^−1^). Although the cotton yield of the bed planting method is highest in the current study, still less than previously reported (Mudassir et al. 2021), the primary reason could be late sowing. However, cotton yield in bed planting is obvious to choose the best method among others. Water productivity is directly proportional to crop yield (Keller 2005). However, plastic mulching may reduce the water productivity as 19.6%, 22.2%, and 14.3% and cotton yield as 19.8%, 23.3%, and 15.6% compared to without plastic film mulching in flat, ridge, and bed planting sowing method. It indicates that plastic mulching practices could reduce water productivity and cotton yield regardless of agronomical practices.

Typically, mulching of polyethylene may cause a higher temperature in soil but is not attributed to higher yield (Moreno et al. 2016). The negative impact of plastic mulching decreases plant growth and yield, increases pest intensification, and compressed the activity of soil microorganisms and soil puddling (Amare and Desta 2021). Alternatively, macro-/microplastic contamination’s soil structural loss is directly attributed to plastic mulching (Huang et al. 2020). Cotton yield is the combined effect of all the yield-influencing factors under particular environmental conditions. Thereby, agronomical practices that enhance the water productivity and stabilize cotton yield need to be implemented as a resilience factor under risk’s climate change. Bed planting (conventional row spacing) of cotton has shown ameliorative plant growth, nutrient uptake, and efficient use of available water resources (Hussain et al. 2021). Thus, bed planting without mulching could be better opt for cotton sowing, water productivity, and cotton yield. Enhancing cotton yield and water productivity could be a major response to the growing demand for cotton.

### HYDRUS-1D model performance

**Fig. 3:**
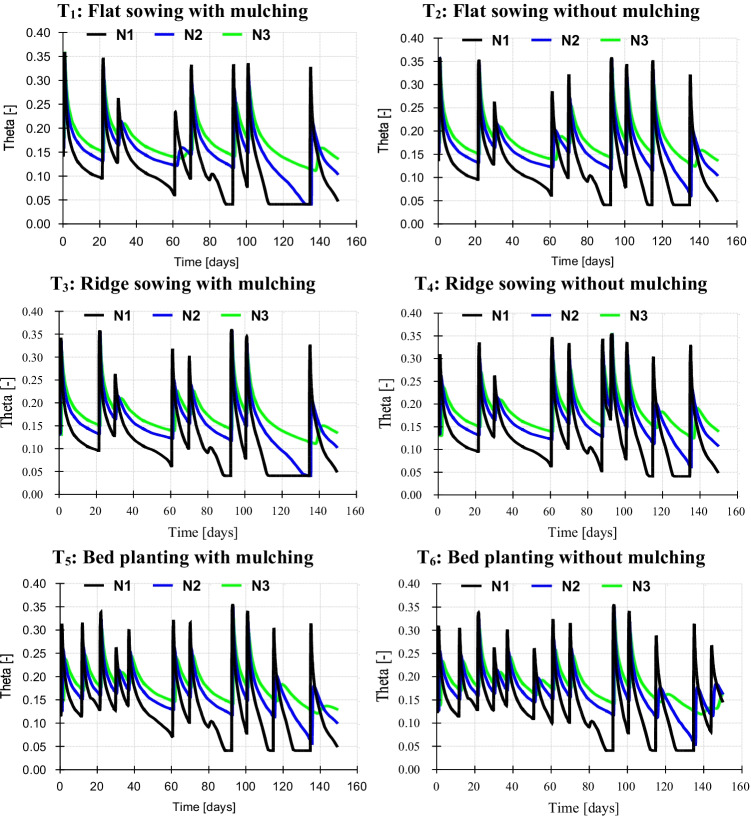
Observation nodes of simulated water content at N_1_ (30 cm), N_2_ (60 cm), and N_3_ (90 cm) under six treatments (T_1_, T_2_, T_3_, T_4_, T_5_, and T_6_)

**Fig. 4:**
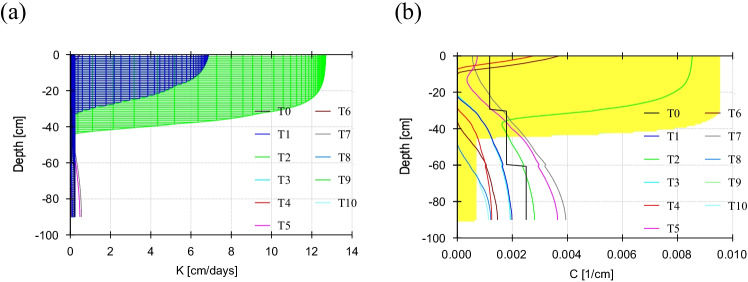
Profile information of soil **a** hydraulic conductivity and **b** hydraulic capacity under ten-time step

Observation nodes on experimental data of pressure heads have shown maximum hydraulic head at 30 cm and minimum hydraulic head at 90 cm depth with a 7-day interval of moisture content of the entire cotton season. Meanwhile, observation nodes of simulated moisture content showed a homogenous pattern (Fig. 3) of moisture content at 30 cm, 60 cm, and 90 cm under six treatments (T_1_, T_2_, T_3_, T_4_, T_5_, and T_6_). Consequently, maximum water content was simulated at 30 cm depth, and minimum water content was simulated at 90 cm depth regardless of the mulching and sowing method. The cotton field has exhibited erratic fluctuations in water content during the season due to surface water and rainfall induction. Nevertheless, the observed data showed a swift decrease in water content than predicted by HYDRUS-1D. This discrepancy in the top layer (30 cm) would be ascribed to more rapid drainage (sandy loam soil) or higher evapotranspiration rates by cotton plants. The model was run under a ten-time step (0–10) and generated a hydraulic conductivity profile and soil layers’ hydraulic capacity. Hydraulic conductivity is a critical parameter to access the water flow into the soil stratum that increased to a certain soil depth then became constant (Fig. 4a). Hydraulic capacity represented the amount of water conveyed through steady and uniform gravity flow (Fig. 4b).

The simulated moisture content (cm^3^.cm^−3^) by HYDRUS-1D at 30 cm, 60 cm, and 90 cm depths corresponded closely to the observed moisture content (cm^3^.cm^−3^) for six treatments. Nevertheless, deviations between simulated and observed moisture content exhibited at certain depths (e.g., 60 cm depth) at treatment T_6_. Moreover, the observed moisture content was higher than the simulated moisture content at 30 cm depth for T_4_ and T_6_ due to the without mulching practice. The robustness of the HYDRUS-1D is evaluated based on *R*^2^, *RMSE*, and *NSE* values, as shown in Table 5. The *R*^2^ and *RMSE* values for the HYDRUS-1D model were ranged between 0.82 and 0.92 and 3.40E − 3 and 6.55E − 5, respectively. Overall, the average *R*^2^ (0.875) and *RMSE* (5.24E − 4) were reliable through the whole soil profile. In addition, *NSE* values (0.85–0.99) explained further the reliability of the model. These results show consistency with previously groundwater recharge flux estimation using HYDRUS-1D under agricultural fields (Turkeltaub et al. 2014; Xi et al. 2016; Boughanmi et al. 2018). Thus, calibrated HYDRUS-1D model has indicated excellent performance, afterward used to simulating the one-dimensional soil water dynamics in irrigated cotton fields for all six treatments.

**Table 5:** Model performance based on *R*^2^, *RMSE*, and *NSE* values between observed and simulated moisture content at different depths and treatments

Treatment	*R*^2^			RMSE			NSE		
	30 cm	60 cm	90 cm	30 cm	60 cm	90 cm	30 cm	60 cm	90 cm
T_1_	0.89	0.86	0.85	1.00E − 3	1.50E − 3	1.50E − 4	0.99	0.84	0.85
T_2_	0.92	0.91	0.87	1.20E − 4	2.50E − 4	4.20E − 5	0.92	0.89	0.87
T_3_	0.85	0.89	0.82	6.55E − 5	1.50E − 4	3.25E − 5	0.86	0.89	0.85
T_4_	0.93	0.88	0.91	6.55E − 5	1.80E − 4	3.22E − 5	0.91	0.84	0.88
T_5_	0.91	0.86	0.87	2.77E − 5	6.55E − 5	1.40E − 3	0.87	0.83	0.86
T_6_	0.89	0.92	0.92	3.40E − 3	5.48E − 4	4.10E − 4	0.86	0.89	0.85

### Groundwater recharge

**Fig. 5:**
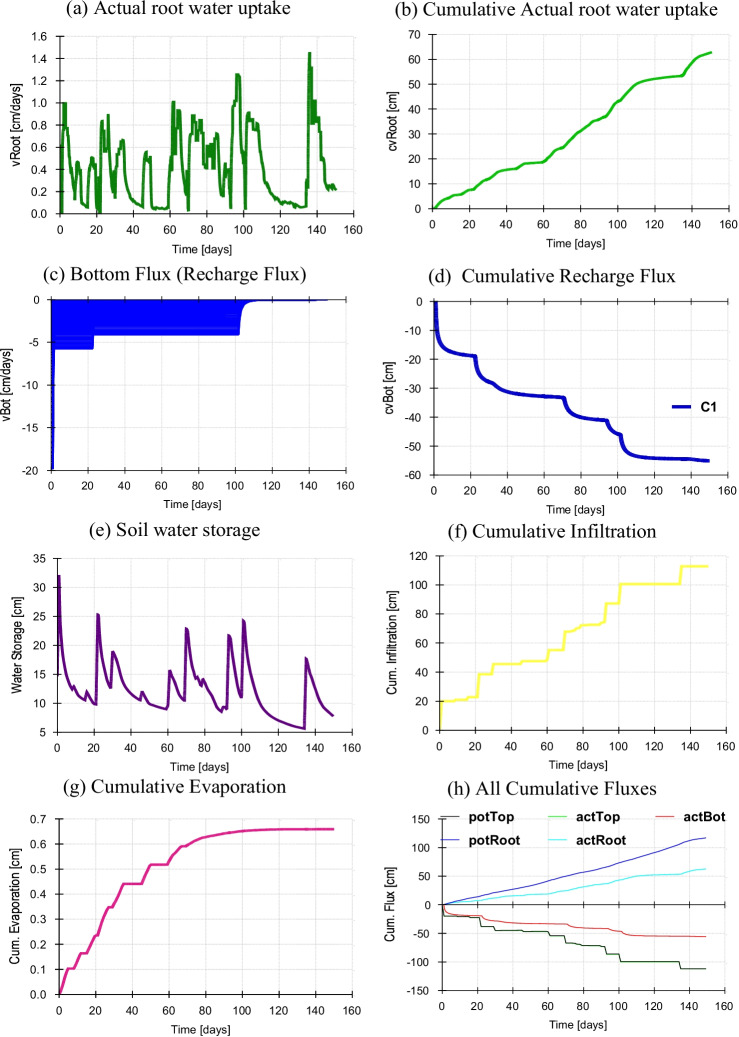
**a–h** Boundary fluxes under flat sowing with mulch

The calibrated HYDRUS-1D model for the irrigated cotton field was run over 150 days at a daily resolution to estimate all fluxes under the flat sowing of the cotton crop with mulch cover in Fig. 5 during the season. Actual root water uptake was minimal during 20–40 days and maximum at 130th after cotton sowing (Fig. 5a). The cotton crop’s cumulative root water uptake is about 62 cm during the 150th day after sowing (Fig. 5b). Bottom flux below the root zone was simulated (Fig. 5c); maximum bottom flux was 5 cm.day^−1^ during the initial stage under T_1_. The cumulative/total recharge flux was 55 cm approximately during the cotton season under T_1_ (Fig. 5d). The amount of water that infiltrates below the root zone attributed to groundwater recharge could be fractional under a 7-day rotational program based on supply instead of demand. Daily soil water storage under T_1_ has a maximum value of 25 cm on the 20th day and a minimum of 5 cm on the 138th day after cotton sowing (Fig. 5e). At this point, the soil moisture content stored could maximize the cotton yield and water productivity (Wang et al. 2020) used by the cotton crop when a deficit of irrigation. The total water depth infiltrated from the ground surface to the subsurface (Fig. 5f), approximately 110 cm during crop duration under T_1_. In this circumstance, the surface runoff is negligible due to small land units or small experimental plots and low rainfall during the simulation period. However, the surface runoff could increase under changing climate scenarios in heavy rain during the monsoon season. Cumulative evaporation from the ground surface is a minimum of 0.6 cm (Fig. 5g) from the soil surface into the atmosphere. All boundary fluxes are shown in Fig. 5h; it reflects that the potential root water uptake is higher than the actual root water uptake.

**Fig. 6:**
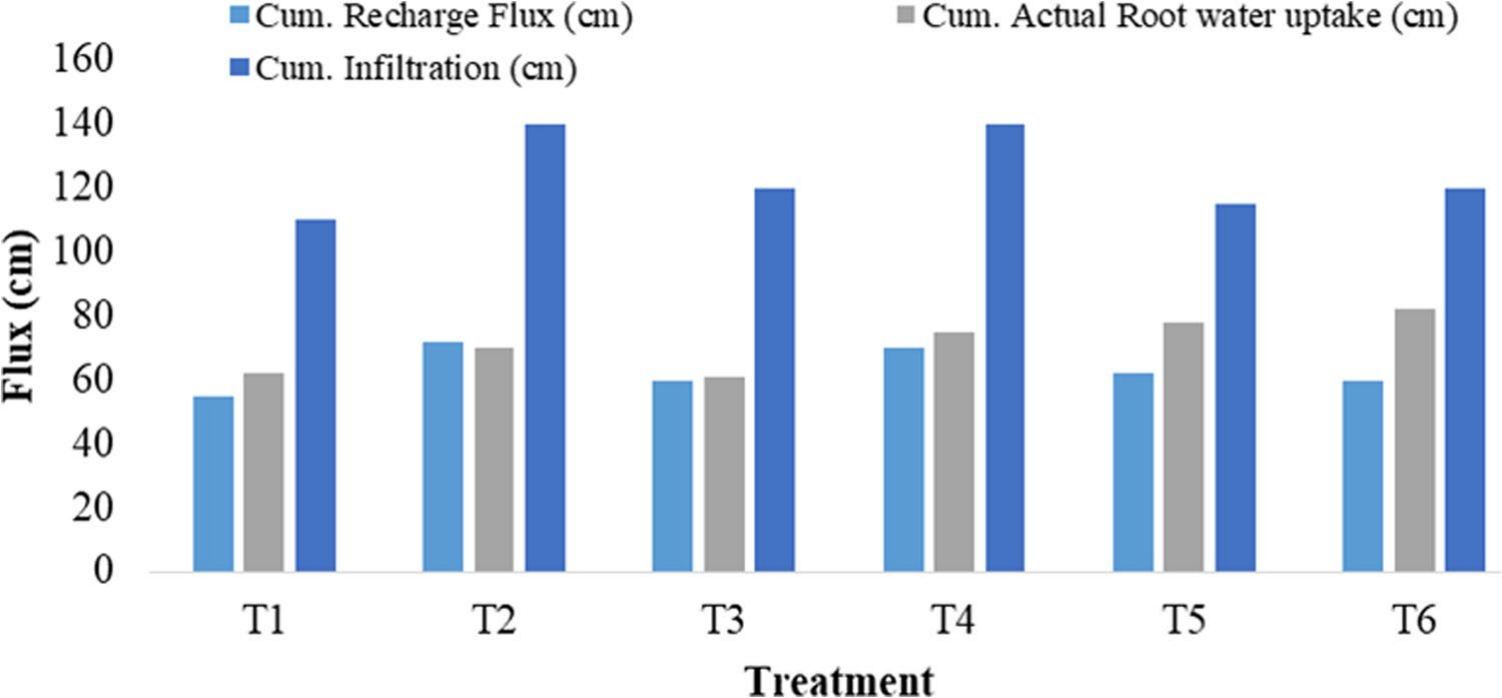
Fluxes simulated using HYDRUS-1D for six treatments

Likewise, all fluxes were estimated for the remaining five treatments T_2_, T_3_, T_4_, T_5_, and T_6_, in the vadose zone of soil using the water budget approach in HYDRUS-1D (Fig. 6). Maximum cumulative recharge occurred under T_2_ (75 cm), followed by T_4_ (70 cm) and T_5_ (62 cm). The crop’s cumulative actual root water uptake was highest under T_6_ (83 cm), followed by T_5_ (78 cm) and T_4_ (72 cm). The estimated cumulative infiltration was observed maximum under T_2_ (140 cm) and T_4_ (140 cm). Plastic mulching of cotton crops under T_1_, T_3_, and T_5_ has reduced soil water content drainage due to climate variability, i.e., rainfall. Meantime, sowing of cotton without plastic mulch has shown more cumulative recharge flux than with plastic mulch. That may have a decisive influence on a water-saving strategy to mitigate water stress in agriculture in rain-fed semiarid crop cultivation areas. Meanwhile, the cumulative water fluxes were reported more in flood irrigation over mulched drip irrigation (Jin et al. 2018).

### Impact of climate change on groundwater recharge

Increasing trends of cumulative actual crop water uptake (90–117.4 cm), cumulative infiltration (118–160 cm), and cumulative evaporation (1.9–2.45 cm) were observed under future changes in climate as compared to 2017 (Fig. 6). That increment could be attributed to rising temperature (2–4 $^\circ{\rm C}$) of the soil top layer (0–20) and increased precipitation frequency and intensity for future climate change (Table 4) (Klik and Eitzinger 2010; Resende et al. 2019). Minimum cumulative infiltration (118 cm) and cumulative actual root water uptake (85 cm) were observed in T_6_ for RCP 8.5 2050 scenario. Treatment T_1_ and T_6_ showed maximum cumulative evaporation (2.45 cm) for the RCP 8.5 2080 scenario. Overall, the actual root water uptake, infiltration, and evaporation trend increased in 2080 than 2050, creating uncertainties in sustainable water resource management (Pool et al. 2021b).

**Fig. 7:**
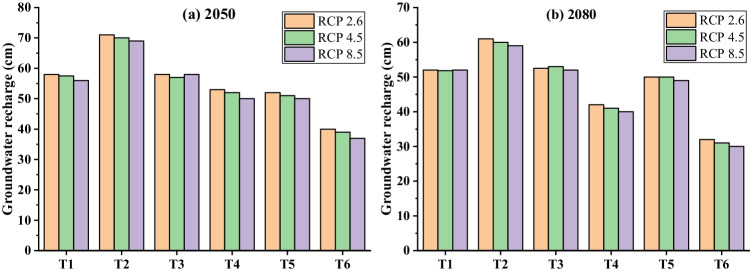
Comparison of RCP 2.6, RCP 4.5, and RCP 8.5 for future (2021–2050 and 2051–2080) cumulative recharge fluxes (cm) under six treatments

Coarse-textured soils of the study area could lower groundwater recharge with a time series of meteorological data (Batalha et al. 2018). The trend of groundwater recharge was accessed for future climate change under six treatments for 2021–2050 and 2061–2080 in Fig. 7. An average (RCP 2.6, 4.5, and 8.5) cumulative recharge flux (cm) for without plastic mulch (T_2_, T_4_, and T_6_) declined 36.53% for 2050 and with plastic mulch (T_1_, T_3_, and T_5_) declined 22.91% for 2080 with a comparison of 2017 (Fig. 6). Meanwhile, groundwater recharge declines an additional 10% in drip irrigation compared to flood irrigation fields (Pool et al. 2021a). However, cover crops provide a service to nitrate pollution mitigation that can reduce groundwater recharge due to higher evapotranspiration than bare soil for water balance (Meyer et al. 2019). The highest recharge was observed 71 cm for 2051–2080 under T_2_ for RCP 2.6 (Fig. 7b), which is relatively high for the semiarid region, indicating a significant increment of precipitation frequency and intensity under future climate change. Groundwater recharge is expected in Southeast Asia, Brazil, and East Africa while increasing over 100 mm.year^−1^ for the Malaysian region (Reinecke et al. 2021). The lowest recharge was 30 cm for 2021–2050 under T_6_ for RCP 8.5 (Fig. 7a). A decline in groundwater recharge could be a potential threat to the socio-ecological balance of the region (Rodríguez-Huerta et al. 2020). Semiarid areas must respond to groundwater dynamics to combat risk from climate change.

Groundwater recharge declined significantly for the cotton sowing method without plastic mulch (T_2_, T_4_, and T_6_). Multilinear regression was conducted to quantify the impact of climate variables on recharge for future changes in climate in Table 6. The correlation coefficient (*r*) value shows weak uphill linear relationships between groundwater recharge and climate variables. Sign of coefficients indicates heterogeneity of groundwater recharge versus climate variable. Results revealed that the model is only bound to elucidate the variation in groundwater recharge ranging from 5 to 23.78% (point to *R*^2^, coefficient of determination) under climate variables. In T_6_-RCP 2.6, climate variables account for only 23.78% of groundwater recharge flux. Precipitation can only contribute up to 4% of recharge flux (Sandoval et al. 2018). In comparison, 76.22% of the variation in recharge flux could be explained by other influential factors, i.e., flood and drought, fate and transport of solute, drainage practice, and soil texture along with soil organic matter. Uncertainty in climate change is significant. An increase and decrease in groundwater recharge can be valid for a specific region per groundwater level (Reinecke et al. 2021).

**Table 6:** Multivariate regression analysis of groundwater recharge in terms of climatic parameters

Treatments and Scenarios	Coefficients					*r*	*R*^2^
	*T*_max_	*T*_min_	*R*_f_	*R*_H_	*W*_S_		
**T**_**2**_**- RCP 2.6_2050**	− 0.4724	0.2867	1.1948	− 0.0531	− 1.231	0.379	0.144
**T**_**4**_**- RCP 2.6_2050**	0.1356	− 0.0758	0.3322	0.0258	− 0.0244	0.2403	0.0577
**T**_**6**_**- RCP 2.6_2050**	0.2260	− 0.2	2.3135	0.0239	0.1416	0.4876	0.2378
**T**_**2**_**- RCP 4.5_2050**	0.0290	− 0.0605	2.7084	0.0572	− 1.0409	0.4807	0.2311
**T**_**4**_**- RCP 4.5_2050**	0.0904	− 0.0931	0.2538	0.0071	0.0143	0.1455	0.0512
**T**_**6**_**- RCP 4.5_2050**	− 0.1618	− 0.0066	0.7273	− 0.0542	− 0.3692	0.2691	0.0724
**T**_**2**_**- RCP 8.5_2050**	0.5207	− 0.3450	0.6784	0.2086	0.7801	0.4579	0.2097
**T**_**4**_**- RCP 8.5_2050**	0.1724	− 0.1463	0.4760	0.0267	0.1245	0.2822	0.0796
**T**_**6**_**- RCP 8.5_2050**	− 0.0034	− 0.0028	1.3989	− 0.0115	− 0.1705	0.2346	0.0550
**T**_**2**_**- RCP 2.6_2080**	0.5037	− 0.1978	1.1592	− 0.1776	− 0.0648	0.2101	0.0841
**T**_**4**_**- RCP 2.6_2080**	0.0904	− 0.0931	0.2538	0.0071	0.0143	0.2777	0.0771
**T**_**6**_**- RCP 2.6_2080**	0.0934	− 0.0069	0.1552	0.1214	− 0.0151	0.1484	0.0520
**T**_**2**_**- RCP 4.5_2080**	0.3669	− 0.3275	1.5760	0.0468	1.0508	0.4046	0.1637
**T**_**4**_**- RCP 4.5_2080**	− 0.7728	0.3510	0.4367	− 0.2107	0.5417	0.4057	0.1646
**T**_**6**_**- RCP 4.5_2080**	0.0631	− 0.1600	0.3921	− 0.0546	0.6086	0.2951	0.0871
**T**_**2**_**- RCP 8.5_2080**	− 0.0909	0.0510	2.1426	0.0417	− 1.1370	0.3936	0.1549
**T**_**4**_**- RCP 8.5_2080**	− 0.5533	0.3666	0.7395	− 0.0521	0.0489	0.4592	0.2109
**T**_**6**_**- RCP 8.5_2080**	− 0.3018	0.7681	0.1489	− 0.0330	− 0.6301	0.3887	0.1511

*p* > 0.05; significant level 95%

In general, for each 1 °C increase in average temperature and 1% increase in precipitation, there is a corresponding decrease of 1.64% and an increase of 0.05% in cotton yield (Li et al. 2021). In Pakistan, the cotton yield fell from 699 to 534 kg.ha^−1^ under climate change, elevating cotton irrigation requirement from 960.1 to 1048.9 mm (Arshad et al. 2019; Abbas 2020; Rehman 2021). In this scenario, reduction in groundwater recharge by 36.53% for irrigated cotton fields indicates that the area is vulnerable to the detrimental impact of climate change. That is threatening to future agricultural and food security and socio-economic development as groundwater pledge a (56% in Pakistan and 76% in the United Arab Emirates) gap in agriculture water demand (Maqbool et al. 2021; Al Tenaiji et al. 2021). Furthermore, sustainable agronomical practices can improve water productivity and increase cotton yield by 8.79% under adaptation measures (Li et al. 2021). However, groundwater recharge could exert additional pressure on country water resources as cotton’s water scarcity footprint is 60% higher than cotton in the Pakistani Punjab (Mikosch et al. 2020). Adopting climate-smart agriculture in water stress regions (arid and semiarid) would combat climate change, water scarcity, and groundwater depletion, enhancing cotton yield by 90 kg.ha^−1^ (Jamil et al. 2021). The current study elucidates two significant facts: first, bed planting has less recharge flux and infiltration and more cotton yield. Second, without plastic mulch, cotton has a greater yield than mulch. Therefore, as future groundwater recharge declined, there is a need to develop and implement effective strategies to maintain groundwater and agricultural development sustainability. In addition, it is equally important to adapt climate change measures for sustainable water resource management to avoid food security risks in the future.

## Conclusion

An experimental study was conducted in a semiarid region to investigate the groundwater recharge flux of irrigated cotton fields under six treatments, i.e., T_1_ (flat sowing with mulch), T_2_ (flat sowing without mulch), T_3_ (ridge sowing with mulch), T_4_ (ridge sowing without mulch), T_5_ (bed planting with mulch), and T_6_ (bed planting without mulch). Water productivity, cotton yield, and the future impact of changing climate on groundwater recharge were investigated. Here is the key finding of the study:

1. Cotton bed planting without mulch showed maximum water productivity (0.24 kg.m^−3^) and the highest cotton yield (1946 kg.ha^−1^). Plastic mulching may reduce the water productivity as 19.6%, 22.2%, and 14.3% and cotton yield as 19.8%, 23.3%, and 15.6% compared to without plastic film mulching in flat, ridge, and bed planting sowing method.
2. The performance of the model was evaluated by *R*^2^ (0.82–0.92), *RMSE* (3.40E − 3–6.55E − 5), and *NSE* (0.85–0.99) that depict good reliability of the model for the assessment of groundwater recharge under the irrigated cotton field. HYDRUS-1D was simulated from sowing to harvesting periods that are 150 days under six treatments. Maximum cumulative recharge was observed 75 cm for T_2_. Cumulative actual root water uptake was highest at 83 cm for T_6_. Cumulative infiltration was observed maximum under T_2_ (140 cm) and T_4_ (140 cm). Plastic mulching of cotton crops under T_1_, T_3_, and T_5_ has reduced soil water content.
3. Using historical data from 1975 to 2005, SDSM was conducted by CanESM2 for the 2050s and 2080s climate scenarios RCP 2.6, 4.5, and 8.5. A temperature rise is expected at 2–4 °C. Meantime, rainfall, relative humidity, and wind speed will decline significantly.
4. Average (RCP 2.6, 4.5, and 8.5) cumulative recharge flux (cm) declined 36.53% in 2050 and 22.91% in 2080 with a comparison of 2017 for without plastic mulch (T_2_, T_4_, and T_6_). Multivariate regression analysis revealed that only a 5–23.78% reduction in groundwater recharge could influence future climate change. There is a weak (positive) relationship between groundwater recharge and climate variables.

Therefore, it is equally important to adapt climate change measures for sustainable groundwater resources to avoid food security risks in the future. It is recommended that the current cotton sowing shall be replaced with bed planting, and mulching practice should be prohibited for greater cotton yield to achieve sustainable development. Further study requires to understand the remaining influencing factor of depleting groundwater recharge.

## Data Availability

Data are available upon request from the corresponding author.

## Footnotes

1 Units of the parameters are interchangeable based on required unit of depth.
